# Moving Beyond ERP Components: A Selective Review of Approaches to Integrate EEG and Behavior

**DOI:** 10.3389/fnhum.2018.00106

**Published:** 2018-03-26

**Authors:** David A. Bridwell, James F. Cavanagh, Anne G. E. Collins, Michael D. Nunez, Ramesh Srinivasan, Sebastian Stober, Vince D. Calhoun

**Affiliations:** ^1^The Mind Research Network, Albuquerque, NM, United States; ^2^Department of Psychology, University of New Mexico, Albuquerque, NM, United States; ^3^Department of Psychology, University of California, Berkeley, Berkeley, CA, United States; ^4^Helen Wills Neuroscience Institute, University of California, Berkeley, Berkeley, CA, United States; ^5^Department of Cognitive Sciences, University of California, Irvine, Irvine, CA, United States; ^6^Department of Biomedical Engineering, University of California, Irvine, Irvine, CA, United States; ^7^Research Focus Cognitive Sciences, University of Potsdam, Potsdam, Germany; ^8^Department of ECE, University of New Mexico, Albuquerque, NM, United States

**Keywords:** EEG, ERP, blind source separation, partial least squares, canonical correlations analysis, representational similarity analysis, deep learning, hierarchical Bayesian model

## Abstract

Relationships between neuroimaging measures and behavior provide important clues about brain function and cognition in healthy and clinical populations. While electroencephalography (EEG) provides a portable, low cost measure of brain dynamics, it has been somewhat underrepresented in the emerging field of model-based inference. We seek to address this gap in this article by highlighting the utility of linking EEG and behavior, with an emphasis on approaches for EEG analysis that move beyond focusing on peaks or “components” derived from averaging EEG responses across trials and subjects (generating the event-related potential, ERP). First, we review methods for deriving features from EEG in order to enhance the signal within single-trials. These methods include filtering based on user-defined features (i.e., frequency decomposition, time-frequency decomposition), filtering based on data-driven properties (i.e., blind source separation, BSS), and generating more abstract representations of data (e.g., using deep learning). We then review cognitive models which extract latent variables from experimental tasks, including the drift diffusion model (DDM) and reinforcement learning (RL) approaches. Next, we discuss ways to access associations among these measures, including statistical models, data-driven joint models and cognitive joint modeling using hierarchical Bayesian models (HBMs). We think that these methodological tools are likely to contribute to theoretical advancements, and will help inform our understandings of brain dynamics that contribute to moment-to-moment cognitive function.

## Introduction

In the neural sciences, we are rarely afforded a one-to-one relationship between neural signals and phenotypic expression of a behavior or disease. It is increasingly common to use computational models to distill the latent features mediating brain-behavior relationships. Indeed, the field of computational psychiatry has emerged to formally address how such latent factors may inform clinically relevant expressions of disease (Montague et al., 2012; Huys et al., 2016). While this approach has leveraged neuroimaging to tie latent features to neural mechanisms, the field of human electrophysiology (electroencephalography, EEG) has been somewhat underrepresented by this approach. The purpose of this review article is to provide a theoretical and methodological manifest to advance the unique virtues of computationally-informed EEG. We first describe approaches to extract features from EEG, including time-frequency decomposition and machine learning approaches. Next we review the extraction of latent features from behavior derived from cognitive models. Finally, we highlight approaches to link these datasets *post hoc* with standard statistical tests, or to derive features jointly using data-driven joint decomposition, or cognitive joint modeling using hierarchical Bayesian models (HBMs).

EEG is commonly known as a useful neuroimaging measure due to its portability, affordability and high temporal resolution. In addition, EEG provides a direct measure of neural activity, since it reflects the aggregate synchronous synaptic activity of hundreds of thousands of radially oriented cortical pyramidal cells (Nunez and Srinivasan, 2006). There is a long history of literature demonstrating relationships between EEG oscillations and cognitive function (Klimesch, 1999; Jensen et al., 2007; Nyhus and Curran, 2010; Harmony, 2013), as well as the relationship between event-related potential (ERP) components and cognition and perception (Regan, 1989; Luck and Kappenman, 2012; Luck, 2014). However, researchers are beginning to focus on other features of EEG such as single trial transient events and non-sinusoidal fluctuations (Jones, 2016; Cole and Voytek, 2017). For example, waveform shapes may differ between different conditions (Cole et al., 2017), and amplitude modulations may be present within only a subset of peaks that follow the steady-state response (Bridwell et al., 2017).

ERP components are presumed to reflect latent computational operations (Luck, 2014), but they remain nebulously defined and are altered by experiment-specific (i.e., modality) and population level (i.e., age) factors, challenging their utility (Donchin et al., 1978). These issues are pointedly stated by Erol Basar in the book *Brain Function and Oscillations* in the following quote:

*“Usually the averaged evoked response is described in terms of several arbitrarily defined components such as peak (wave) latencies and wave magnitudes. These arbitrarily defined components depend generally upon the location of the recording electrode, behavioral state or sleep stage of the subject under study, and upon the nature of the stimulating signal. Therefore, the interpretation of these arbitrarily defined components is very difficult …”* (Basar, 1999)

It is widely observed but rarely formally acknowledged that event-related EEG activities reflect canonical neural operations that are predictably modulated within spatio-temporal windows. ERP components are only revealed by averaging across individual trials and subjects—these components (and their corresponding interpretations) may not hold at the individual subject or individual trial level (Rousselet and Pernet, 2011; Luck, 2014). For example, Gaspar et al. (2011) demonstrate that ERP responses to faces and noise are reliable across repeated measurements of the same subject, but that the individual subject averages differ among each other and with the group average. Thus, the emphasis on component peaks which appear following trial and subject averaging may detract from the ability to detect EEG features at the single trial or single subject level, and may limit the ability to relate those features with behavior.

Moreover, components are oftentimes defined by latent cognitive constructs (i.e., attention, working memory; Donchin and Coles, 1988; Folstein and Van Petten, 2007), which may not be effective characterizations of underlying neural computations. For example, it is not likely that different ERP components map onto the chapter titles within Psychology textbooks. In order to address these issues, we focus on deriving features from EEG which are not dependent upon the identification of canonical ERP component peaks (and their interpretations). For example, the processes which contribute to these stimulus and subject averaged responses may be decomposed based on their temporal frequency, and single trial data can be transformed to a lower dimensional representation using machine learning tools such as independent component analysis (ICA), representational similarity analysis (RSA), general linear modeling (GLM) and deep learning, as described in subsequent sections. We think that these methodological advancements are likely to contribute to theoretical advancements, and help to advance this field beyond a focus on ERP components.

**Figure 1:**
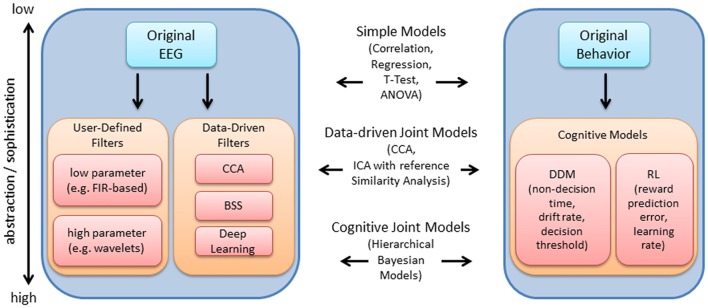
Overview of electroencephalography (EEG) and behavioral processing steps reviewed, and approaches to integration. The original EEG data is depicted within the upper left. The arrow points to a series of processing steps which aim to extract features relevant to the experiment. These processing steps are generally comprised of user-defined (i.e., pre-selected) temporal filters with few or many parameters, or data-driven filters such as canonical correlation analysis (CCA), blind source separation (BSS) and deep learning. Within this article, we review time-frequency decomposition (with wavelets), various BSS approaches and deep learning approaches. These processing steps are useful for enhancing the signal at the single-trial level, which improves the ability to detect relationships between EEG and behavior. Using a similar depiction on the right, the original behavioral data (e.g., hit rate, false alarm rate, reaction time) may be used to derive latent measures of cognitive function (e.g., using drift diffusion models (DDM), or reinforcement learning (RL) models as examples). Measures at various levels of abstraction/sophistication within EEG and behavior may be combined using various approaches reviewed, including simple statistical models, data-driven joint models and cognitive joint models, as indicated in the middle of the plot.

Figure 1 provides an overview of the EEG and behavioral processing steps discussed within this review. The original EEG or behavioral signal represents the lowest level of abstraction, i.e., is closest to the original measurement, while various processing steps may be subsequently implemented to abstract from the original measurement using models that are justified mathematically, biologically, or both (in contrast to the data-driven focus within the EEG literature, behavioral measures are often directly linked with computational mechanism and cognitive theory via cognitive modeling; Turner et al., 2016). Within Figure 1, while the level of abstraction increases from top to bottom within the blocks for EEG and behavior, the level of sophistication of the approaches to link each dataset increases from top to bottom (as depicted within the center of the blocks in Figure 1). At the lowest level of sophistication, the EEG and behavioral features are derived independently and then linked *post hoc*, using standard statistical tests. At a higher level of sophistication, EEG and behavioral features are extracted independently and links are identified by decomposing the features within a common framework, i.e., joint data-driven decomposition such as joint-ICA (jICA) or multiset Canonical correlation analysis (CCA; i.e., “late fusion” approaches described within Dahne et al., 2015). Each level provides increasingly more information to the data fusion approach (Calhoun and Sui, 2016). Further, EEG features may contribute to the estimation of cognitive model parameters in parallel with direct measures of behavior (i.e., by specifying an explicit relationship between EEG features and behavioral features). HBMs have been implemented to accomplish linking in this manner, allowing EEG and behavioral features to jointly contribute to estimates of higher level cognitive processes (e.g., Frank et al., 2015; Nunez et al., 2017).

By appropriately constructing models, these analyses should strive for understanding cognitive function at the single trial level within healthy and patient populations. Within the sections that follow, the EEG processing steps are described first, followed by the extraction of latent variables from direct measures of behavior. Then, simultaneous data-driven decomposition and joint approaches to integrate these measures are reviewed. Importantly, many of these methods can be implemented using publicly available toolboxes (see Table 1).

**Table 1:** Select publicly available toolboxes for electroencephalography (EEG) analysis.

Toolbox	Notable functions	Citation(s)	Link
EEGLAB	BSS	Delorme and Makeig (2004)	http://sccn.ucsd.edu/eeglab
EEGIFT	Group BSS	Calhoun et al. (2001) and Eichele et al. (2011)	http://mialab.mrn.org/software/eegift/
FIT	Joint ICA; CCA + Joint ICA; Parallel ICA	Calhoun et al. (2009) and Calhoun and Sui (2016)	http://mialab.mrn.org/software/fit
EP Toolkit	PCA	Dien (2010)	http://sourceforge.net/projects/erppcatoolkit/
LIMO EEG	Hierarchical Linear Modeling of EEG	Pernet et al. (2011)	http://github.com/LIMO-EEG-Toolbox/limo_eeg

*BSS, blind source separation; ICA, independent component analysis; CCA, canonical correlation analysis; PCA, principle component analysis*.

## Overview of Extracting Features From EEG

### Selecting User-Defined Features With Time-Frequency Filters

**Figure 2:**
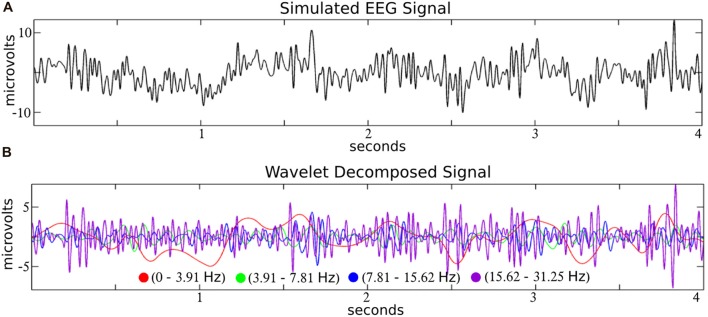
Time-frequency decomposition of simulated EEG with wavelets. A simulated EEG signal (**A**; from the SIMEEG toolbox http://mialab.mrn.org/software/simeeg/index.html) was decomposed into time courses which correspond approximately to the delta (0–3.91 Hz), theta (3.91–7.81 Hz), alpha (7.81–15.62 Hz) and beta (15.62–31.25 Hz), EEG frequencies **(B)**. Wavelet coefficients were estimated using the discrete wavelet transform (DWT) implemented in MATLAB (http://www.mathworks.com) with the wavedec and wrcoef functions (biorthogonal spline mother wavelet; bior3.9; dyadic decomposition; 5 levels; Adapted from Bridwell et al., 2016).

Like a prism to sunlight, time-frequency filters decompose a time-series input into a dimension-expanded representation of frequency-specific temporal activities. In the context of EEG, filters like wavelets decompose the signal into biologically reasonable frequency bands (see Figure 2), and we focus on wavelets here as a representative example of a time-frequency filter.

Wavelet convolution operates by computing the similarity over time between the input signal and a template short-time frequency-specific oscillation (motivating the diminutive “let” after the word “wave” in “wavelet”). Morlet wavelets are constructed by multiplying a complex exponential carrier with a Gaussian window. A family of Morlet wavelets may include 50 frequency-specific sine waves (e.g., 1–50 Hz), each tapered (i.e., convolved) with a Gaussian function (Lachaux et al., 2002). This would reveal a dimension-expanded 50-frequency by time matrix of activity from the input signal.

To understand this approach, it is important to know that convolution in the temporal domain is mathematically equivalent to multiplication in the frequency domain. This means that while wavelet convolution is a useful concept to understand how signal similarity underlies this transformation, the same output is usually computed by the much faster procedure of multiplying each of the Fourier coefficients for the input signal and the wavelet followed by reconstruction of the time-series via inverse Fourier transform. At its core, the wavelet procedure has some mathematical equivalence with other techniques for deriving time-frequency representations like the short time Fourier or band-passed Hilbert transform (Bendat and Piersol, 2010). However, these are oftentimes inexact replications, as parameters like filter width alter the output in subtle but meaningful ways. The pervasiveness of the Fourier transform across techniques offers an excellent way to double-check one’s data processing for hidden bugs by comparing outputs derived via wavelet vs. filter-Hilbert techniques (Le Van Quyen et al., 2001; Yuan and Luo, 2012).

The dimension-expanded representation includes both the phase angle of the frequency-specific signal at each temporal moment (instantaneous phase) as well as the amplitude of this signal (usually squared to derive power). This decomposition is tremendously important for continued specification of the neural features that underlie cognitive operations. If multiple trials of an event are associated with consistent phase angles, the event is likely re-setting ongoing phase dynamics or instantiating a phase-dependent neural process (Makeig et al., 2002). This has strong implications for the nature of underlying neural networks that are created and dissolved over various time scales (and frequencies). Brain regions that are phase consistent are likely using phase dynamics to structure temporal windows for neurons to simultaneously fire and form a transient neural network (Fries, 2005). To clarify some unfortunately common terminology that appears in the field, wavelets do not reveal “oscillatory” activity, nor are oscillations necessary inputs for wavelet decomposition. Any time-series signal can be decomposed into a time-frequency representation; the repeating nature of the signal is irrelevant.

The process of wavelet convolution (or equivalently, Fourier coefficient multiplication) is conceptually and procedurally quite simple. However, many free parameters are critical for successful usage such as frequency range selection, taper dynamics, signal length and selection of an appropriate baseline to obviate the non-linear distribution of power between frequencies (i.e., the 1/f nature of EEG spectra). An interested scientist can find a full description of each of these issues as well as MATLAB code in the book *Analyzing Neural Time Series Data* (Cohen, 2014).

### Blind Source Separation

Time-frequency decompositions (e.g., wavelets, discussed in the previous section) can emphasize signals that correspond to a distinct frequency band, removing artifacts that typically fall outside biologically plausible frequencies (e.g., <1 Hz changes in skin impedance, high frequency muscle artifacts, and 50/60 Hz electrical line current). However, broadband artifacts and artifacts which overlap in frequency with biological oscillations are more problematic for this filtering approach. These scalp signals are superimposed with various sources of noise, including eye blinks, eye movement, muscle tension, heartbeat, AC electrical line current, and skin potentials. In addition, some sources of noise influence neural signals directly, e.g., when the content of visual stimuli changes with eye movements or blinks. Thus, various blind source separation (BSS) techniques have been applied to derive data-driven filters which disentangle these signal mixtures.

BSS approaches have been developed and implemented for matrix decomposition (Onton et al., 2006) or higher order (*N* ≥ 3 dimension) tensor decomposition (Cong et al., 2015) of EEG. These approaches provide an improved separation of EEG signal from EEG signal, or EEG signal from noise. Temporal ICA and principle component analysis (PCA) are two common EEG data reduction approaches with relative advantages and disadvantages that depend on the degree in which the data aligns with the methods assumptions (Bugli and Lambert, 2007; Dien et al., 2007). PCA derives a set of latent variables which are uncorrelated, while temporal ICA decomposes the data into a linear mixture of temporally independent sources and their associated loadings or mixing matrix (i.e., scalp topography; Makeig et al., 1997; Hyvarinen et al., 2001; Stone, 2004). Temporal ICA has been particularly popular since assumptions of the approach are consistent with the linear mixture of independent cortical electrical potentials that occurs across the scalp due to volume conduction. And temporal ICA is particularly robust as a pre-processing step in EEG artifact removal, since eye blinks and eye movement artifacts are independent and non-Gaussian sources which contaminate the EEG. These artifactual sources may be removed before converting the data back to the original channel space (Jung et al., 2000; Makeig et al., 2004; Onton et al., 2006).

Temporal ICA and PCA have demonstrated utility in decomposing the multiplexed ERP response into distinct ERP peaks (for temporal ICA, see Onton et al., 2006; Bridwell et al., 2014, 2015, for PCA see Bernat et al., 2005), which likely leads to a more accurate estimate of the amplitudes of distinct ERP components than the original superimposed response. However, these models suffer from similar caveats as many of the modeling approaches discussed—they require extensive user attention and parameter selection (e.g., PCA components may differ depending on how many are selected for rotation, the type of rotation and how the data are conditioned; Kayser and Tenke, 2003; Dien et al., 2005).

In general, artifacts are readily separated from EEG sources with temporal ICA due to their non-Gaussian distribution (Hyvärinen et al., 2010). However, temporal ICA seems ill equipped at separating non-artifact sources, motivating the use of alternative BSS approaches for decomposing different EEG oscillations (e.g., EEG collected in the absence of an explicit task), including algorithms designed to decompose EEG oscillations explicitly and those that decompose real or complex valued EEG spectra (Anemüller et al., 2003; Bernat et al., 2005; Hyvärinen et al., 2010; Porcaro et al., 2010; Nikulin et al., 2011; Shou et al., 2012; Bridwell et al., 2016). These approaches are well suited for decomposing the broad EEG spectra into functionally distinct frequency bands, including separating the alpha band (8–12 Hz) into its low and high frequency sub-bands (Niedermeyer, 1997; Nunez et al., 2001; for an example, see Bridwell et al., 2013).

Various extensions to BSS have been developed to address the issue of determining which BSS sources correspond across subjects, including approaches which decompose the aggregate group EEG data (Kovacevic and McIntosh, 2007; Congedo et al., 2010; Eichele et al., 2011; Cong et al., 2013; Lio and Boulinguez, 2013; Ponomarev et al., 2014; Ramkumar et al., 2014; Huster et al., 2015; Huster and Raud, 2018; Labounek et al., 2018). Within temporal Group ICA implemented in EEGIFT^1^, sources are estimated at the aggregate group level and individual subject sources are recovered by back-reconstruction (Calhoun et al., 2001; Calhoun and Adali, 2012). This approach effectively “filters” the individual subject data by the source activity that is robust at the group level. However, the emphasis on the aggregate group dataset introduces issues in generating reliable decompositions in cases where there are topographic differences across subjects (Huster et al., 2015). Nevertheless, Group ICA is particularly advantageous in cases where individual trials are analyzed, as demonstrated by Bridwell et al. (2014), where single trial ERP amplitudes were correlated with single trial measures of prediction error or surprise. Within further studies, these single trial amplitudes would provide useful input measures to simple and joint models which integrate EEG and behavior (e.g., as discussed in the sections on Single Trial Regression and HBMs).

### Deep Learning

**Figure 3:**
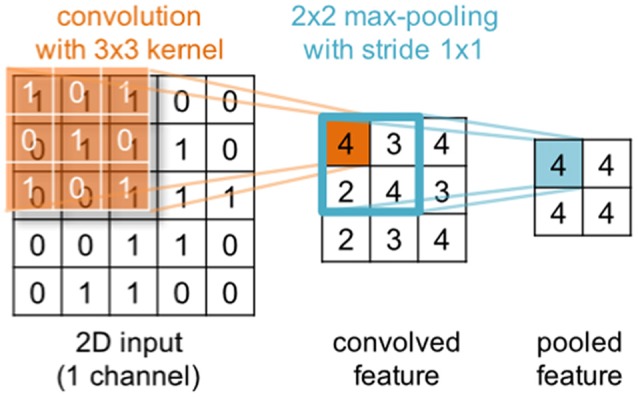
Illustration of a convolution operation with a single 2d 3 × 3 kernel (orange) applied to a 2d 5 × 5 input. In a convolutional layer, the input is convolved with trainable filters. Generally, input and kernels may have different shapes and span multiple channels (not shown here). Further, multiple kernels can be applied in parallel within the same convolutional layer. The convolution is commonly followed by an element-wise application of a non-linear transformation (not shown) and optionally a pooling step (blue) that aggregates neighboring output values.

**Figure 4:**
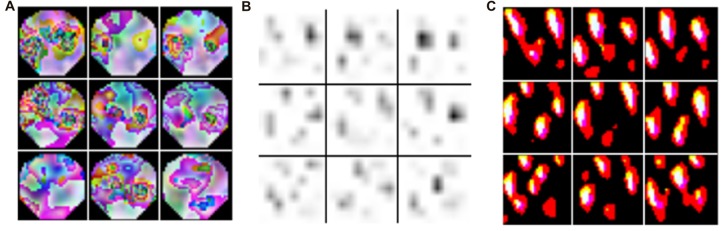
Topographic features derived from kernels in recurrent-convolutional neural networks. The input EEG with the highest activation across the training set is indicated in **(A)**. Feature maps were derived from the kernel (#122 in the 3rd stack output) and plotted in **(B)**, and back-projected topographies were computed using deconvnet in **(C)**. The figure is modified from Bashivan et al. (2016) and reproduced with permission.

Deep learning is a subfield of machine learning that during the last decade has gained substantial popularity by breaking benchmark records in various domains (such as computer vision, automatic speech recognition, natural language processing and bioinformatics). The term deep learning was coined as a reference to deep artificial neural networks (ANNs) with more than one hidden (i.e., non-input or -output) layer of neurons. Most notably, these techniques are able to learn complex non-linear functions that transform raw signals into feature representations or derive probability distributions over latent variables. The latter can be achieved through probabilistic graphical models like restricted Boltzmann machines (RBMs) or deep belief nets (DBNs). Popular approaches for the former are convolutional and recurrent neural networks—CNNs and RNNs respectively (for a review in the context of neuroimaging, see Plis et al., 2014). In a convolutional layer, the input is convolved with trainable filters as illustrated in Figure 3, whereas recurrent connections feed the layer output back into earlier layers as additional input. All these techniques support unsupervised training for signal modeling and learning high-level patterns as well as supervised training for discriminative tasks such as classifying EEG based on the recorded trial type or upon collected behavioral measures. Importantly, the approach can incorporate the entire spatial and temporal structure, and the outputs of the kernel may be visualized as topographic feature maps (Figure 4; Bashivan et al., 2016).

A common approach for unsupervised training of ANNs which goes back to the 1980s is the auto-encoder (Bourlard and Kamp, 1988). Such a network consists of an encoder and a decoder. The encoder transforms input data into an internal representation whereas the decoder computes a reconstruction of the input from this representation. The whole network is trained by minimizing the reconstruction error for some training dataset. In order to make this task non-trivial and force the auto-encoder to learn meaningful features that represent the data well, a representational bottleneck is commonly required—for instance, limiting the number of neurons in the inner-most layer (structural bottleneck), adding noise to inputs or activations, or sparsity regularizations. Auto-encoders can be stacked to incrementally learn higher-level features.

With respect to supervised learning approaches, a neural network pre-training technique for learning discriminative features from stimulus-driven EEG signals was described by Stober (2017). Demanding that encodings of two trials from the same class are more similar to each other than to encoded trials from other classes, a respective encoder pipeline was trained that improved the signal significantly even across subjects. This tuple-based training approach is especially suitable for small datasets and measures with a low signal-to-noise ratio (SNR), such as EEG.

In applying deep learning to EEG datasets, there are a few important considerations. Investigators should be careful in instances where unrealistically high accuracy is observed, as this is usually an indicator of a flawed experiment. For instance, the way the data was recorded and split into subsets for training and evaluation could have introduced artifacts that were exploited by the classifier. This so-called “Horse” phenomenon has, for instance, been described by Sturm (2014). Another potential cause for flawed results are software bugs such as mistakes in indexing trials or accidentally duplicating trials that result in overlapping training and evaluation data.

## Overview of Estimating Latent Variables From Behavior

The previously discussed EEG processing steps derive useful features which may be subsequently related to behavior. Within this section, we review approaches to integrate latent cognitive variables for linking with EEG within subsequent steps, which differs from the typical focus on observable, experimenter-defined objective variables. For example, categorical variables (e.g., stay vs. switch trials) are used to pool trials into groups across which the neural signal is then compared (Collins et al., 2014), or continuous variables (e.g., the amount of conflict) can be used for single trial analysis of the EEG signal (Cohen and Cavanagh, 2011). This categorical approach can be improved by implementing cognitive modeling (i.e., computational modeling of behavior) techniques to estimate latent variables on a trial by trial basis, and subsequently relating these measures with EEG.

Computational models of behavior usually propose a mechanistic or algorithmic description of the computations that may be happening in the brain to support behavior. These models usually have parameters (e.g., drift rate or learning rate, see below for details), that quantitatively modulate the computations made by the model. Model fitting techniques allow us to infer the parameters that are most likely to give rise to the observed behavior. Then, given a set of parameters for a model, it is also possible to obtain the latent variables that are part of the models’ computations, and thus that putatively are the underlying variables needed to account for the observed behavior.

Thus, cognitive modeling may provide two types of benefits to relate behavior and EEG signal. First, fitting computational models to behavioral data allows researchers to extract model parameters that are putatively related to mechanisms underlying behavior; these parameters may then capture variability (between conditions or individuals) better than raw behavior would. Second, model fitting also allows researchers to extract latent variables that putatively reflect the computations supporting behavior. These variables may then be better candidates to reflect the trial-by-trial neural signal. In the next paragraphs, we give an overview of the computational modeling process with these two target benefits, and focus on two key examples: drift diffusion modeling (DDM) for perceptual decision making (Ratcliff, 1978; Ratcliff and McKoon, 2008), and reinforcement learning (RL) modeling (Sutton and Barto, 1998).

**Figure 5:**
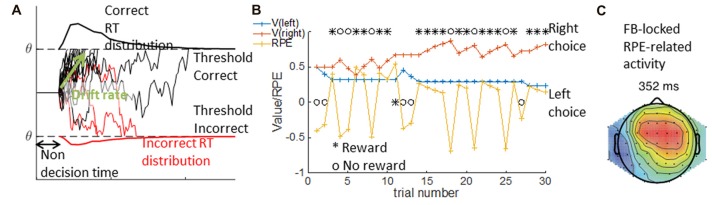
DDM and RL Model. In the DDM in **(A)**, single traces show multiple examples of simulated noisy accumulation of evidence to correct (black) or incorrect (red) decisions. The resulting distribution of reaction times are plotted for correct (top) and incorrect trials (bottom). The DDM relies on three main parameters—the non decision time, the threshold q indicating the bounds to which evidence is accumulated, and the drift rate indicating the rate of evidence accumulation. In the RLM depicted in **(B)**, an example of a sequence of 30 learning trials is given where the left choice is rewarded with probability *p* = 0.2, and the right with probability *p* = 0.8. Given a choice and reward history (black), the computational model provides the inferred underlying changes in expected value for each option (V, red and blue traces), and the inferred reward prediction error (RPE, yellow). The latent variables from modeling can be used to analyze trial by trial voltage. Within **(C)** activity over mid-frontal electrodes is correlated with RPEs from correct trials (modified from Collins and Frank, 2016).

The DDM is a special case of a sequential sampling model (SSM) we focus on here. The DDM is a used to account simultaneously for accuracy and reaction time observations in binary perceptual decision tasks, such as the random dots motion task (e.g., Roitman and Shadlen, 2002). Specifically, the DDM formalizes decision as a noisy accumulation of evidence to one of two bounds; it assumes that once the decision variable reaches the bound, the corresponding choice is made. The DDM is usually parameterized with three parameters: non-decision time, drift rate and threshold. The non-decision time reflects a fixed period of time during which no information is accumulated; mechanistically, it may include both initial perception latency and motor command latency after the decision is made. The drift rate reflects the rate at which information is accumulated, or the strength of each new piece of evidence. The threshold indicates the level the evidence should reach prior to a decision being taken. Other parameters are sometimes included in the DDM to better capture behavior; for example, a bias term may be needed to capture participants’ tendency to select one option more than another (see Figure 5A).

Simulations of the DDM can show how changing the parameters modifies performance, and reaction times for different conditions (e.g., correct vs. incorrect trials; Ratcliff and McKoon, 2008). For example, with a higher threshold, more evidence accumulation is required before reaching a decision, which is less likely to happen due to noise; thus, the observed behavior is more conservative: fewer errors with longer reaction times. Slower drift rates produce a qualitatively similar pattern, but quantitatively different. We thus need a quantitative method to disentangle the role of different parameters in the observed behavior of participants. The model fitting procedure computes a measure of fit, the ability of the model to predict the distributions of choices and reaction times across the two conditions for a given set of parameters; it then attempts to maximize this fit measure to find the set of parameters that best explain the behavior. Fitting can be done separately for different subjects or conditions, and fit parameters can then be related to neural signal—for example, frontal theta power and DDM threshold (Cavanagh et al., 2011).

Model fitting can also provide inferred trial-by-trial latent variables on which decisions rely. For example, in a learning task where participants learn to select the best out of two options using probabilistic feedback (Frank et al., 2004), the inferred learned value of each option at each time point is a continuous variable of interest that is not directly observable. RL models assume that participants track an estimate *V*_t_ of the value of different options at each time *t*, and that they update this estimate when they obtain a reward *r*_t_ by incrementing it by a proportion of the reward prediction error (RPE), the difference between the observed reward and the predicted reward: *rpe* = *r*_t_ − *V*_t_. By how much the estimate is incremented is controlled by the learning rate parameter α, such that the new estimate at time *t* + 1 is *V*_t+1_ = *V*_t_ + *αrpe*. The choice between two options depends on the estimated values, and can be controlled by a noise parameter (e.g., the softmax inverse temperature β; see Figure 5B).

The best parameters for a participant can be obtained by a similar model-fitting procedure to the one described for the DDM. Once the parameters are inferred, researchers can deduce a sequence of latent variables defined by the computational model, the fit parameters and participants’ choice and reward history. For example, in a probabilistic learning task, we can extract the sequence of expected values and RPEs that best explain participants’ choices. These latent variables can then be linked to the neural signal on a trial by trial basis (e.g., Collins and Frank, 2016, and Figure 5C), thus giving insight into the computational mechanisms underlying behavior.

While using computational models for analyzing behavior and linking it to the neural signal can be extremely helpful, it also has many potential pitfalls that should be carefully kept in mind, and a number of methods can mitigate them (Nassar and Frank, 2016). The most important step is the validation of the model. It is impossible to be sure that a given model is a good mechanism for a cognitive process of interest, however, it is important to ensure that it is a reasonable candidate. A relative measure of a model’s goodness includes comparing its fit to other candidate models. Quantitative comparisons should take into account model complexity, as more complex models are more likely to overfit the data. A more absolute measure of a model’s goodness of fit requires posterior predictive checking: it is important to simulate a model that would result from the best-fit parameters and ensure that the pattern of simulated behavior is a qualitative match to participant performance.

## Data Fusion: Linking EEG/ERPs and Behavior in An Exploratory/Data-Driven Manner

In the sections that follow, we highlight approaches to link EEG data and behavioral data without imposing cognitive modeling constraints on their relationships. Single trial regression identifies associations between the two measures based upon their linear relationships, while the data-driven approaches (i.e., data-driven joint models) link EEG and behavior based upon the particular assumptions of the BSS model. These approaches do not incorporate an explicit link to cognitive parameters as the joint cognitive models discussed within the subsequent section (see *“Linking EEG/ERP and Behavior With Latent Variable Estimation (Hierarchical Bayesian Models)” section*), but nevertheless hold considerable utility in revealing the relationship between EEG responses and behavior.

### Single Trial Regression

Trial-by-trial correlations between neural signals and behavior offer the most stringent correlational test of brain-behavior relations. Yet, it is arguably more common to see brain-behavior correlations computed between, rather than within individuals. While this can account for important individual differences, it does not offer a mechanistic test of how the brain facilitates behavior.

As an example, consider the question about how self-generated error signals motivate behavioral correction. The Error-Related Negativity (ERN) is a robust EEG marker of motor errors of commission (Falkenstein et al., 1991; Gehring et al., 1993), and early reports showed that it predicted slower response times following the error: an important manifest indicator that may indicate the application of cognitive control (Laming, 1979; Gehring et al., 1993). Subsequent studies contributed inconsistent evidence for a predictive relationship between MCC control signals and behavioral adjustments (see Weinberg et al., 2012). The ambiguity, however, reflected an over-focus on between-subject examinations and a failure to examine within-subject trial-to-trial brain-behavior relationships.

A meta-analysis of 20 between-subjects studies revealed a significant meta-effect between error signals and post-error slowing, yet five within-subjects studies revealed a significantly larger relationship (Cavanagh and Shackman, 2015). Importantly, these between subjects findings don’t tell us if the ERN is actually linked to subsequent behavioral adjustment: it could be the case that, for example, a personality dimension like anxiety simply makes people more cautious in general.

In statistical terminology, within-subject single trial regressions can be considered a Level 1 analysis and between-subjects brain-behavior regressions are a Level 2 analysis. These can be modeled together using mixed linear modeling (Singer and Willett, 2003), hierarchical linear modeling (see “*Linking EEG and Behavior With Latent Variable Estimation (Hierarchical Bayesian Models)” section)*, or by using the regression weight of the Level 1 analysis as a first-order statistical summary for input into standard General Linear Models (i.e., as implemented in toolboxes such as SPM, Fieldtrip and LIMO EEG). This simple approach has been used to show that people with Generalized Anxiety Disorder indeed have different single trial brain-behavior relationships than controls, suggesting that (Level 2) individual differences influence (Level 1) mechanisms for instantiating cognitive control (Cavanagh et al., 2017).

In practice, the low signal to noise features of EEG lead to limited resolution of single trial activities. This can be bolstered by the approaches discussed within this review, including spatial and temporal filtering (see Debener et al., 2005), but users often apply non-parametric methods like Spearman’s rank correlation coefficient in order to diminish the influence of outliers and non-linearity. Single trial multiple regression can be computed in MATLAB with ample protection against outliers using the robustfit function, which uses robust regression to down-weight outliers. Alternatively, LIMO EEG implements first and second level GLM’s using robust statistical tools which emphasize effect size and which do not require preselection of peaks or components.

### Similarity Analysis

One of the issues that arises in identifying relationships between unique datasets is the difference in the nature and dimensionality of each measure. We highlight RSA as a promising method for transforming different datasets into a common representational space prior to assessing their similarity (Kriegeskorte et al., 2008). Of note, RSA has been used to identify relationships among diverse types of neuroimaging datasets, such as EEG and magnetoencephalography (MEG; Su et al., 2012, 2014), MEG and fMRI (Cichy et al., 2014, 2016), ERP’s and fMRI (Salmela et al., 2018), or intracranial EEG (iEEG) and fMRI (Zhang et al., 2015). Relationships among neuroimaging measures and behavior (Wardle et al., 2016), neuroimaging measures and competing models (Kaneshiro et al., 2015), neuroimaging measures and stimulus features (Su et al., 2014), may also be examined.

Within RSA, similarities among responses within a dataset are first derived by computing a matrix of similarities between responses across separate conditions or stimuli. This matrix, termed the representational dissimilarity matrix (RDM) may be constructed from pairwise correlation distances, pairwise classification accuracies (Cichy et al., 2016), or from confusion matrices from multi-class classifications (Kaneshiro et al., 2015). The RDM matrix represents a complexity reduced representation of the original dataset which preserves information within the original data space (i.e., by correlating fine-grained spatial or temporal activations, as opposed to the loss of information of conventional averaging; Kriegeskorte et al., 2006). By relating different RDM matrices, RSA provides a framework for identifying relationships among neuroimaging measures and behavioral data or computational models (Kriegeskorte et al., 2008).

**Figure 6:**
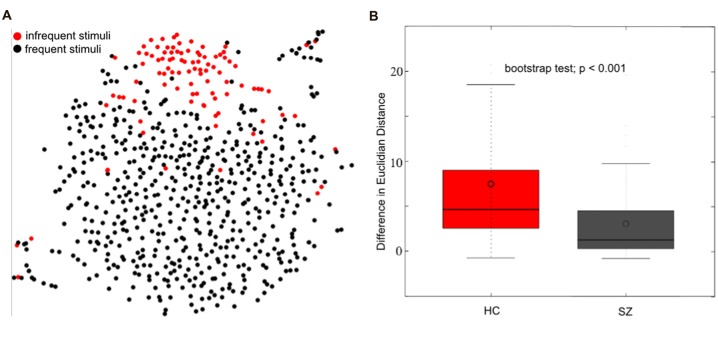
Single trial event-related potential (ERP) projection using t-Distributed Stochastic Neighbor Embedding (t-SNE). t-SNE projects multi-dimensional datasets into a lower dimensional space for visualization. A high dimensional 654 (98 infrequent stimuli + 556 frequent stimuli) × 12224 (64 electrodes × 191 time-point) matrix of single trial ERP’s was projected to two dimensions using t-SNE (single healthy subject) in **(A)**. The single-trial ERP responses to infrequent auditory oddball stimuli (in red) reasonably separate from the single-trial ERP responses to frequent auditory stimuli (in black), motivating the use of t-SNE for visualizing multi-electrode single-trial ERP’s. The separation between frequent and infrequent stimuli was quantified as the average Euclidian distance between frequent and infrequent stimuli minus the average distance among infrequent and infrequent stimuli. A boxplot of the distribution of differences is indicated in **(B)** for healthy controls (HC) and patients with schizophrenia (SZ). These results indicate a better separation among frequent and infrequent stimuli among HC than SZ (bootstrap test; *p* < 0.001; modified from Bridwell et al., 2018).

To visualize differences between conditions, the pairwise distances among the elements within an individual RDM may be projected to two or three dimensions using multidimensional scaling (MDS; Edelman et al., 1998), and the hierarchical structure may be visualized using hierarchical clustering (Dubes and Jain, 1980). These visualization tools hold considerable promise for preserving the high-dimensional structure of these datasets, which we demonstrate using t-Distributed Stochastic Neighbor Embedding (t-SNE; van der Maaten and Hinton, 2008; Figure 6). t-SNE emphasizes the local structure of data points as opposed to the global structure, emphasized by MDS. Thus, there are different advantages to each dimension reduction approach depending on the structure of the dataset that the researcher seeks to highlight (and the nature of the data; Rieck and Leitte, 2015; Kruiger et al., 2017).

While t-SNE has been a useful visualization tool for fMRI and MRI datasets (Mwangi et al., 2014; Du et al., 2015; Mahfouz et al., 2015; Panta et al., 2016), it has been underutilized within EEG. Within Figure 6A, we demonstrate the t-SNE projection (perplexity = 25; PCA dimensions = 50; tolerance = 0.0001) of single trial 64 channel ERP oddball data collected on a single healthy subject. The trials containing frequent stimuli (in black) reasonably separate from the trials containing infrequent stimuli (in red). This separation was quantified by computing the average Euclidian distance among infrequent and frequent stimuli minus the average distance among infrequent stimuli. Using this measure, we observed a greater separation between the two trial types among healthy controls (HC; *N* = 58) than among individuals diagnosed with schizophrenia (SZ; *N* = 58; Figure 6B; bootstrap test; *p* < 0.001; for participant and experiment information, see Bridwell et al., 2014). These results demonstrate that the information within single-trial EEG’s may be meaningfully projected to a two-dimensional representation with t-SNE (Bridwell et al., 2018). These findings provide motivation for using visualization tools which consider the high dimensional structure of the data. However, careful consideration should be given to approaches better suited to continuous data such as EEG, and different ways to quantify the separation of points in the lower dimensional space (e.g., Mahalanobis distance; Mahalanobis, 1936).

### BSS Decompositions Incorporating Multiple Datasets

BSS extracts meaningful information from large datasets in a data-driven manner (for a review in the context of neuroimaging, see Silva et al., 2016). Focusing on a lower dimensional EEG dataset reduces the number of statistical relationships between EEG measures and external variables, which alleviates the possibility of being overly conservative in correcting for multiple statistical comparisons. This is advantageous when identifying relationships between EEG responses and behavior, as well as when examining associations between EEG and other high dimensional datasets, such as fMRI (Calhoun et al., 2009; Eichele et al., 2009; Bridwell and Calhoun, 2014).

Beyond integrating information from multiple modalities in parallel, many approaches have been developed and implemented to extract concurrent fluctuations from multiple measures in a joint decomposition, including simultaneous decompositions of EEG’s and fMRI (Martínez-Montes et al., 2004; Calhoun et al., 2006, 2010; Moosmann et al., 2008; Edwards et al., 2012; Mijović et al., 2012, 2014), EEG spectra and structural MRI (sMRI; Soh et al., 2015), or EEG spectra, fMRI and sMRI (Sui et al., 2014), often with applications to clinical populations (Calhoun et al., 2009; Adali et al., 2015; Calhoun and Sui, 2016). Including all data within a multivariate multimodal decomposition isolates complex relationships between measures that may be more difficult to observe with more conventional tests, improving the ability to observe relationships between multimodal networks and behavior.

Instead of examining *post hoc* associations between neuroimaging measures and behavior, a more integrated approach is to decompose neuroimaging and multivariate behavioral profiles at the same time (Liu et al., 2008; Groves et al., 2011; Calhoun and Sui, 2016). Since these are two fundamentally different types of data (e.g., with different dimensions), a more flexible version of jICA, termed parallel-ICA, has been particularly promising. Parallel-ICA imposes constraints on the two datasets during decomposition such that correlated components are decomposed together (for details see Liu et al., 2008; Pearlson et al., 2015). For example, Meier et al., 2012 isolated a sub-component of the “resting state” network (RSN) that co-varied with behavior in a sustained attention task, and a sub-component of the left inferior frontal gyrus that co-varied with behavior during a memory task (Meier et al., 2012).

Decomposition algorithms have also been developed to decompose neuroimaging data into components which maximally differ based on a reference measure, such as single nucleotide polymorphism (SNP) allele frequencies, cognitive state estimates, or behavioral measures. The Source Power Comodulation (SPoC) approach, for example, is effective at identifying components which correlate with a target variable (e.g., correct responses, reaction time, or stimulus features), especially low SNR responses (Dähne et al., 2014). As opposed to the standard data-driven decomposition of components, decomposition with reference imposes constraints on the decomposition such that sources are extracted which meaningfully differ based upon an external variable of interest (e.g., Liu et al., 2012).

Multi-dataset decompositions with reference have also been implemented recently by Qi et al. (2018). The authors applied “multi-site canonical correlations analysis with reference + jICA” to decompose fMRI, sMRI and dMRI data using working memory performance as a reference (Qi et al., 2018). The approach (implemented in the FIT toolbox^2^) holds considerable promise by providing a joint framework for identifying information from multiple modalities based on cognitive and behavioral differences among populations. And future studies may incorporate EEG data within these decompositions to identify differences associated with behavioral measures such as reaction times or percent correct, or latent behavioral measures such as DDM parameter estimates.

### Partial Least Squares

Predictive brain-behavior relationships can be established using partial least squares (PLS) regression analyses (N-way toolbox, Andersson and Bro, 2000) of EEG. PLS is well established in neuroimaging studies (McIntosh et al., 1996; McIntosh and Lobaugh, 2004) as a method to address the problem of multicollinearity in regression models where a very large number of independent variables derived from brain data are used to model a few behavioral variables. PLS regression models estimate orthogonal components (similar to PCA) which maximize the covariance between behavioral data and the EEG data. More specifically, the PLS components are based on the optimization of a least-squares fit of a partial correlation matrix between the EEG data and dependent variables of interest (Andersson and Bro, 2000). Although PLS components lead to optimal regression models using only a few components, they are susceptible to overfitting, since the component structure is determined by the covariance with behavioral data. Thus, PLS models (as well as CCA models) must be validated by testing the prediction of new data not used in estimating the model (Huang et al., 2011; Kang et al., 2016).

Although PLS has been more extensively used in neuroimaging, a number of studies have made use of PLS models to relate EEG and evoked potential properties to individual differences in behavior. In studies of attention, PLS models have been applied to steady-state visually evoked potentials (SSVEP) responses to flickering signals and noise to account for how individual differences in the deployment of spatial attention can predict accuracy data (Krishnan et al., 2013). Similar models of the SSVEP have also been used to assess the optimal attentional filters in detection of biological motion (Hasan et al., 2017). In studies of motor learning, PLS models have been used with resting state EEG coherence to demonstrate that the functional connectivity of motor cortex can predict short-term motor learning (Wu et al., 2014). In studies of stroke patients, PLS models have shown that EEG coherence with motor cortex in the resting state can predict both motor impairment and improvement over a period of rehabilitation (Wu et al., 2015, 2016).

### Canonical Correlation Analysis

CCA is conceptually similar to PLS (Rosipal and Krämer, 2006), except that in PLS linear structure is assumed as hidden (i.e., “latent”) variables while CCA is a more nonparametric approach to finding weights that produce maximum correlation (for additional comparisons between CCA and PLS, see Sun et al., 2009; Dahne et al., 2015).

CCA transforms two multi-dimensional datasets such that the resulting variates are maximally correlated across datasets and uncorrelated within each dataset (Hotelling, 1936). The application of CCA to joint datasets consisting of EEG and behavioral or cognitive measures is limited, so we highlight select studies here which have used multi-subject extensions of CCA to identify covariations between EEG data and other neuroimaging measures such as fMRI, and sMRI (for an example linking brain connectivity patterns and demographic and psychometric measures, see Smith et al., 2015).

Multi-subject extensions of CCA (termed multimodal CCA, jCCA or mCCA) have been developed using ERP time courses and fMRI spatial maps as features (Li et al., 2009; Correa et al., 2010a). Unlike jICA, which is also implemented on multi-subject multi-modal datasets, mCCA allows a separate mixing matrix for each modality and does not emphasize independence among the multimodal sources. Thus, mCCA is suitable for operating on diverse data-types with differing dimensionalities. The relationships between multi-modal feature loadings and behavioral variables such as percent-correct, confidence ratings, and average reaction time may then be examined (Correa et al., 2008, 2009, 2010a,b). Further extensions of mCCA to single trials will be useful for identifying associations in concurrently recorded ERP and fMRI datasets, and the loadings of these multi-modal variates may be related to corresponding single trial behavioral measures.

## Linking EEG and Behavior With Latent Variable Estimation (Hierarchical Bayesian Models)

Within this section, we focus on combining EEG and behavior for simultaneously estimating latent cognitive measures. Hierarchical Bayesian modeling (HBM) of human cognition is one of the most powerful methods to integrate EEG and behavior, since these datasets are linked with respect to the *cognitive* function specified by the model and shared relationships are estimated simultaneously. The hierarchical Bayesian modeling (HBM) framework is ideally suited for the joint analysis of multiple modes of data (Lee, 2011; Turner et al., 2013). In addition, the EEG data can also provide new and additional information about the cognitive process that cannot be discerned with just behavior alone. This flexible framework can inform building and testing theoretical models of the relationship of electrical observations from the human cortex (EEG), human cognition and human behavior.

Bayesian inference refers to underlying probability theory and methods used to obtain conclusions about data (for an entertaining introduction to Bayesian inference see Etz and Vandekerckhove, 2017). Hierarchical modeling refers to the mathematical procedure of assuming statistical relationships between multiple levels of data description. Hierarchical modeling often yields better estimates of parameters due to shrinkage, a phenomenon whereby parameters are better estimated (and data are better described) because hierarchical relationships enforce similarity across similar parameters. For instance, the condition-level mean accuracies in an experimental task could statistically describe observed subject-level mean task accuracies through a normal distribution, and yield more predictive estimates of future subject-level ability (see Lee and Wagenmakers, 2014 for an introduction to HBMs). HBM also allows discovering complex relationships between multiple data types within cognitive neuroscience (Wiecki et al., 2015; Turner et al., 2016) by allowing simultaneous estimation of posterior distributions of multiple parameters. Fitting procedures produce samples from probability distributions that display knowledge (i.e., “uncertainty”) about parameter estimates and thus certainty about the effects of cognition or EEG data in specific theoretical models.

**Figure 7:**
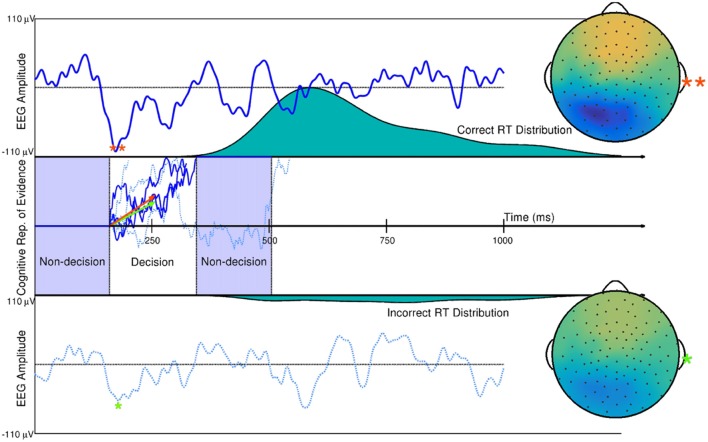
Single trial ERP influence on DDM parameters. Two trials of a subject’s spatially weight-averaged ERP are shown (top and bottom panels) along with simulations of this subject’s *cognitive representation of evidence* (middle panel) that are derived from RT distributions. The 10th and 90th percentiles of this subject’s single-trial drift rates (within-trial average *evidence* accumulation rates in a Brownian motion process as assumed by the DDM) are shown as the orange and green vectors. Results from hierarchical Bayesian modeling suggested that single-trial N200 amplitudes (peaks and spline-interpolated scalp maps denoted by the orange and green asterisks) influence single-trial drift rates (i.e., one latent cognitive parameter that describes the time course and latency of a decision). Using fitted parameters from real data, the larger drift rate is a linear function of the larger single-trial N200 amplitude (**), while the smaller drift rate is a linear function of the smaller N200 amplitude (*). The three dark blue evidence time courses were generated with the larger drift rate (orange vector) which is more likely to produce faster reaction times (where one path describes the time course of the example *decision time* and subsequent remaining *non-decision time* in the Middle panel). The three dotted, light blue evidence time courses were generated with the smaller drift rate (green vector) which is more likely to produce slower reaction times. True Brownian motion processes were estimated using a simple numerical technique discussed in Brown et al. (2006). Further explanation of the simulation and model fitting exists in Nunez et al. (2017).

Using the DDM of quick decision-making as an example, single-trial estimates of evidence accumulation rate during quick decision making and non-decision time (time in milliseconds of a human reaction time not related to a decision) have been obtained using hierarchical Bayesian modeling with ERP amplitude estimates on single trials, time-locked to the onset of visual stimuli. Such work is described in Nunez et al. (2017). It was found that ERP measures described trial-to-trial differences in visual encoding time (a component of non-decision time during reaction time) and trial-to-trial differences in evidence accumulation rate, as described by trial-level estimates of the drift parameter (Figure 7). EEG correlates of additional cognitive processes, such as visual attention, can also add inference about the overall human cognitive process when used in combination with behavioral modeling. Nunez et al. (2015) found evidence that differences in experimental participants’ attention (both visual noise suppression and visual signal enhancement) as measured by SSVEPs related to some specific differences in participants’ cognition during decision-making.

Modern software allows HBM to be easily created, built and tested with both behavioral and EEG data using multiple types of Markov Chain Monte Carlo (MCMC) sampling techniques (see Table 2). Although still being developed and improved, JAGS (Plummer, 2003), Stan (Carpenter et al., 2017), and PyMC3 (Salvatier et al., 2016) are all recommended tools for these steps. These programs allows users to build almost any cognitive hierarchical models they choose. For example, these programs can readily be adapted to HBM of EEG and behavior using RL models. However, cognitive neuroscientific theory must be further developed to learn useful combined generative models of observed cortical dynamics, cognition, and human behavior. Specific examples of these programs in use include (1) HDDM: python functions that can perform linear regression between calculated EEG signals on single-trials and parameters of DDMs of accuracy and reaction time data (Wiecki and Frank, 2013); (2) Hawkins et al. (2017) created R code and examples for sampling from hierarchical drift-diffusion models (HDDM) with neural inputs^3^; and (3) MATLAB and JAGS example code performing some HDDM analyses with single-trial EEG inputs can be found at https://github.com/mdnunez/mcntoolbox (Nunez et al., 2017).

**Table 2:** Publicly available toolboxes for (hierarchical) Bayesian parameter fitting.

Toolbox	Programming language	Citation	Link
JAGS	Base software and Interfaces for Python, R, and MATLAB	Plummer (2003)	http://mcmc-jags.sourceforge.net/
Stan	Interfaces for Python, R, MATLAB, and Julia	Carpenter et al. (2017)	http://mc-stan.org/
PyMC3	Python	Salvatier et al. (2016)	https://github.com/pymc-devs/pymc3

## Conclusion

Neuroimaging research is focused on understanding the links between neural activity and observable behavior. This knowledge will help us understand how individual differences in the brain contribute to individual differences in cognitive processes. EEG is low cost, portable and provides millisecond resolution of brain activity. Thus, it is a promising tool for examining neural measures at the time scales of cognitive function. In order to relate EEG features with behavior, it will be important to obtain reliable single-trial estimates of EEG features using advanced processing and machine learning tools described within this review, and to move beyond the conventional focus on evoked components derived after averaging across trials and subjects. In addition, the accumulation of EEG depositories through data sharing and quantitative extensions beyond binary comparisons will also help advance the field. The resulting single trial EEG measures may be correlated with direct or latent measures of behavior *post hoc*, decomposed jointly with behavior, or integrated with behavior to derive estimates of latent variables which represent cognitive function. In doing so, we hope to further our understanding of brain dynamics that contribute to moment-to-moment cognitive function.

## Author Contributions

DAB, JFC, AGEC, MDN, RS, SS and VDC wrote the manuscript and approved the final version.

## Conflict of Interest Statement

The authors declare that the research was conducted in the absence of any commercial or financial relationships that could be construed as a potential conflict of interest.

## Footnotes

^1^http://mialab.mrn.org/software/eegift/

^2^http://mialab.mrn.org/software/fit

^3^https://osf.io/ws3fn
